# Ophthalmomyiasis Externa: A Report of Three Cases

**DOI:** 10.4274/tjo.70456

**Published:** 2015-10-05

**Authors:** Cem Sundu, Erdem Dinç, Umut Can Kurtuluş, Özlem Yıldırım

**Affiliations:** 1 Mersin University Faculty of Medicine, Department of Ophthalmology, Mersin, Turkey; 2 Aşkım Tüfekçi State Hospital, Clinic of Ophthalmology, Adana, Turkey

**Keywords:** Ophthalmomyiasis externa, keratitis, cyclopentolate

## Abstract

Three patients were admitted to our clinic with symptoms of conjunctivitis. On ocular examination, moving larvae were seen in the conjunctival sac. All of the larvae were immobilized by topical cyclopentolate and removed mechanically under topical anesthesia. The patients healed without any complications after the treatment. Physicians should consider ophthalmomyiasis externa in patients presenting with similar symptoms, especially in areas with high numbers of livestock. Otherwise the diagnosis can be missed.

## INTRODUCTION

Myiasis is the infestation by fly larvae or maggots. While most cases involve the skin, more rarely the eyes, nasal passages, paranasal sinuses and urogenital tract may also be affected. Ocular involvement is seen in less than 5% of cases.^1^ Ophthalmomyiasis is typically seen in farmers and shepherds in rural areas, though it may also affect individuals outside these professions. The condition is most often caused by Oestrus ovis and it exists in two forms, external and internal.^2,3^ External ophthalmomyiasis involves the bulbar or palpebral conjunctiva; internal ophthalmomyiasis involves globe penetration. While the external form is self-limiting, the internal form may lead to severe vision loss. The aim of this study is to discuss three cases of external ophthalmomyiasis and their treatment.

## CASE REPORTS

### Case 1

**Figure 1:**
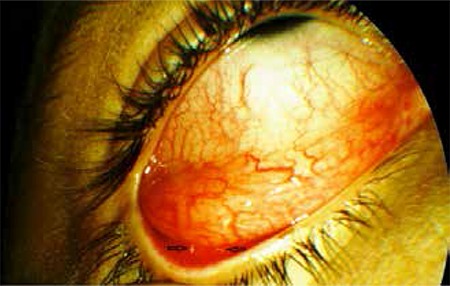
Two larvae are visible on the conjunctival surface of the lower eyelid on the temporal side (black arrows)

A 13-year-old male patient presented to our clinic with complaints of redness, tearing and eyelid swelling in the left eye beginning one day earlier. He reported passing a herd of sheep and goats and an unidentified foreign body striking his eye. Best corrected visual acuity (BCVA) in both eyes was 10/10. Slit-lamp examination revealed motile larvae on the palpebral and bulbar conjunctiva (Figure 1). One drop of 1% cyclopentolate (Sikloplejin®, Abdi İbrahim, Turkey) was applied to the left eye; 10 minutes later, under topical anesthesia with 0.5% proparacaine (Alcaine®, Alcon, Belgium), 6 immobilized larvae were mechanically removed. Following the procedure, the patient was treated with topical lotoprednol (Lotemax®, Bausch+Lomb, USA) drops four times daily and topical lomefloxacin (Okacin®, Novartis, Switzerland) drops four times daily. Examination one week later was normal.

### Case 2

**Figure 2:**
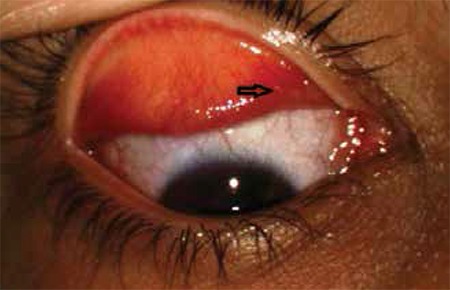
A larva is visible on the conjunctival surface of the upper eyelid on the nasal side (black arrow)

A 21-year-old male patient presented to our clinic with complaints of redness, tearing and blurred vision in the right eye beginning two days earlier. He reported being hit in the right eye with an unknown foreign body while riding a motorcycle and that his primary health care provider had diagnosed bacterial conjunctivitis and prescribed topical antibiotic. BCVA was 10/10 in both eyes. Slit-lamp examination revealed motile larvae in the patient’s right palpebral and bulbar conjunctiva (Figure 2). One drop of 1% cyclopentolate (Sikloplejin®, Abdi İbrahim, Turkey) was applied to the right eye; 10 minutes later, under topical anesthesia with 0.5% proparacaine (Alcaine®, Alcon, Belgium), 7 immobilized larvae were mechanically removed. Following the procedure, the patient was treated with topical lotoprednol (Lotemax®, Bausch+Lomb, USA) drops four times daily and topical lomefloxacin (Okacin®, Novartis, Switzerland) drops four times daily. Examination one week later was normal.

### Case 3

A 55-year-old male patient presented to our clinic with complaints of redness and tearing in this right eye beginning one week earlier. It was learned that he worked with livestock and that 3 days earlier he had been examined by a different ophthalmologist who had removed larvae from his eye. BCVA was 10/10 in both eyes. Slit-lamp examination revealed motile larvae in the right palpebral and bulbar conjunctiva. Furthermore, an area of corneal infiltration was seen in the upper cornea on the 1 o’clock line. One drop of 1% cyclopentolate (Sikloplejin®, Abdi İbrahim, Turkey) was applied to the right eye; 10 minutes later, under topical anesthesia with 0.5% proparacaine (Alcaine®, Alcon, Belgium), 5 immobilized larvae were mechanically removed. Following the procedure, in addition to closure therapy the patient was treated with topical moxifloxacin (Vigamox®, Alcon, USA) every hour, artificial tears, and ketorolac (Acular LS®, Allergan, Ireland) four times daily. At examination one week later, we observed that the epithelial defect had closed and the area of infiltration had healed uneventfully.

## DISCUSSION

Ophthalmomyiasis due to Oestrus ovis was first described in 1947.^4^ Although myiasis in humans is rare, it is seen more frequently in regions where poor hygiene conditions prevail and where sheep and goat husbandry is common.5 The majority of reported cases are in Middle Eastern countries; though rare, cases do occur in Turkey.^1^ The phenomenon occurs more frequently in spring and summer.^5^

Ophthalmomyiasis externa may manifest clinically with symptoms of classic conjunctivitis, pseudomembranous conjunctivitis, blepharoconjunctivitis, punctate keratitis and keratouveitis.^6,7^ The cases in our report all presented with classic conjunctivitis. However, the second patient was diagnosed with bacterial conjunctivitis and treated accordingly, yet his symptoms showed no improvement. For this reason, thorough slit-lamp examination should be performed on patients with similar symptoms, especially in areas with high livestock densities. There are reports in the literature of cases of external ophthalmomyiasis accompanied by keratitis, similar to our third case.^7^ In addition to slit-lamp examination for larvae, other accompanying symptoms should not be overlooked and the treatment should be adjusted as required.

Another important point is the mechanical removal of the larvae. Being highly motile, the larvae can easily escape notice, resulting in lack of symptom improvement. The third patient in our case series had a similar experience when some of the motile larvae were overlooked and not removed initially. For this reason, a second examination should be performed a short time after the initial mechanical removal to locate and remove other larvae that may have been missed. Furthermore, the application of 1% cyclopentolate 10 minutes prior and the topical anesthetic used reduce larval motility, which both facilitates their removal and decreases the likelihood that they will escape detection. There are reports in the literature of using 4% cocaine hydrochloride treatment due to its anticholinergic effect, but to our understanding cyclopentolate has also been used in similar cases.^8^ Cyclopentolate also exhibited effects similar to cocaine when used in the cases in the current study. If obtaining cocaine presents difficulties, the much more easily attainable cyclopentolate can be used in similar cases. There are also reports of using systemic ivermectin to treat cases of nasal involvement secondary to ophthalmomyiasis externa.^9^ Ivermectin may also be used topically.^10^ One of the challenges of mechanically removing larvae is that they are able to attach tenaciously to the conjunctiva using hook-like structures in and around their head. For this reason, the use of forceps facilitates the removal process.

Topical antibiotic and steroid application after treatment can suppress inflammation and prevent secondary infections. Furthermore, in cases with accompanying keratitis such as our third patient, the treatment regime should be adjusted as necessary. With proper cleaning and treatment, results are very positive.

In summary, external ophthalmomyiasis should be kept in mind in the differential diagnosis of patients presenting with conjunctivitis and a thorough slit-lamp examination including the inner eyelids should be conducted to ensure the diagnosis is not overlooked.
